# Dietary Pattern or Weight Loss: Which One Is More Important to Reduce Disease Activity Score in Patients with Rheumatoid Arthritis? A Randomized Feeding Trial

**DOI:** 10.1155/2022/6004916

**Published:** 2022-04-29

**Authors:** Alireza Sadeghi, Mojtaba Tabatabaiee, Mir Ali Mousavi, Seyedeh Neda Mousavi, Somayae Abdollahi Sabet, Nooshin Jalili

**Affiliations:** ^1^Department of Internal Medicine, Vali-e-Asr Hospital, Zanjan University of Medical Science, Zanjan, Iran; ^2^Department of General Surgery, Ayatollah Mousavi Hospital, Zanjan University of Medical Sciences, Zanjan, Iran; ^3^Zanjan Metaboilc Diseases Research Center, Zanjan University of Medical Sciences, Zanjan, Iran; ^4^Department of Nutrition, School of Medicine, Zanjan University of Medical Sciences, Zanjan, Iran; ^5^Department of Community Medicine, School of Medicine, Social Determinants of Health Research Center, Zanjan University of Medical Sciences, Zanjan, Iran

## Abstract

**Objectives:**

Herein, disease activity score 28 (DAS 28) was compared between patients with rheumatoid arthritis (RA) receiving the Mediterranean dietary pattern (MD) and low-fat diet. *Subjects/Methods*. Overweight and obese RA patients aged 15–75 y participated in this randomized feeding trial. Participants were randomized to MD (*n* = 51) and low-fat high-carbohydrate diet (*n* = 53) for 12 weeks. The control group followed their regular diet (*n* = 50). Participants completed the form of tender and swollen joint counts before the study enrollment and after 12 weeks to compute DAS 28.

**Results:**

Weight loss was not statistically significant between the MD and LF-HC groups. DAS 28 significantly decreased in MD compared to the LF-HC group (*p*=0.02) and controls (*p*=0.001). Adjusting for the baseline variables, MD reduced DAS 28 by 76% (95% CI = −0.45, −0.2; *p*=0.03) after 12 weeks of intervention. The baseline serum ESR level showed 99.8% effect on DAS 28 score at the end (95% CI = 0.014, 0.035; *p* < 0.001).

**Conclusions:**

The MD showed beneficial effects on DAS 28 compared to the LF-HC diet in patients with RA, regardless of weight loss. It is a better dietary choice for pain reduction in patients with RA. The trial is registered at Iranian Registry of Clinical Trials (IRCT20200929048876N2).

## 1. Introduction

Rheumatoid arthritis (RA) is characterized by joints involvement and systemic features with destruction of the cartilage and bone due to the involvement of autoimmune or inflammatory pathways [1]. Age, gender, genetics, and some environmental factors are the main risk factors of RA. The aims of RA treatment are reducing joint inflammation and pain in patients along with preservation of function and form of joints. The first-line of disease management are the conventional synthetic disease modifying antirheumatic drugs (cs-DMARDs) and adding a biological drug (b-DMARD) only in the case of therapeutic failure [2]. The DMARDs include methotrexate, sulfasalazine, D-penicillamine, and hydroxychloroquine [3]. All of these drugs have side effects, and patients have discomfort feelings with them.

Studies proposed that the dietary pattern, macronutrient, and micronutrient have a fundamental role in disease risk and progression [4–9]. Mediterranean diet (MD) was introduced as the healthiest dietary pattern for the risk of RA occurrence and progression, as wells as its activity. The MD pattern has differences in various cultures [10–12]. Religious omits some of the essential components of MD in some countries [11]. Therefore, the MD guidelines have differences in various countries, and more studies are needed to reach the beneficial effects of this dietary pattern in patients with RA. On the other hand, bodyweight control is a primary advice in all diseases [13, 14] due to the negative impact on disease activity remission and efficacy of therapy processes. However, the effects of MD are not compared with the low-fat diet on disease severity adjusted for bodyweight reduction in patients with RA in a Muslim population with religious restrictions. Disease activity score was compared between obese and overweight patients with RA receiving MD and low-fat diet on a weight loss program.

## 2. Materials/Subjects and Methods

### 2.1. Study Design and Patient Enrollment

In this randomized single-blinded feeding trial, 129 patients with RA participated. Disease was diagnosed based on the American College of Rheumatology criteria [15]. Overweight and obese patients (25 < BMI < 35 kg/m^2^), aged 15–75 y, without joint replacement history, and with the informed consent were included. Patients with inflammatory or chronic disorders were excluded. Patients under oral corticosteroid therapy, smokers, and heavy drinkers did not participate. Patients on a certain diet and whom reluctant to participate were excluded. The Ethical Committee of Zanjan University of Medical Sciences (IR.ZUMS.REC.1398.425) approved the present trial and registered on 09/10/2020 at Iranian clinical trials website under code number: IRCT20200929048876N2. All procedures were performed according to the best practice guidelines on publication ethics: a publisher's perspective [16].

### 2.2. Dietary Interventions

At the baseline, dietary intake was analyzed by three-day food diary (two regular days and one holiday). Then, RA patients were randomly assigned to the tree dietary groups based on the block randomization method. Diets based on 28 kcal/kg/day were determined. In MD, 35% of total calorie was provided from fasts; however, this portion was 20% in the low-fat group. Protein percentage was equal in the both dietary groups (15%). The remaining percentages were allocated to carbohydrates. Patients on MD were allowed to consume 150 g of red meat per month. Olive and canola oils were recommended as the main source of dietary fat. Participants were encouraged to consume one serving of legume and nut each day, as well as consume low-fat dairies. Mercury-free fish oil supplements were consumed two times per week. The control group followed their regular diet for 12 weeks.

### 2.3. Outcomes

The disease activity score 28 (DAS 28) questionnaire was completed for each patient. This questionnaire consists of twenty-eight tender (Ritchie Articular Index; RAI) and swollen joint count (SJC) scores including the same joints: shoulders, elbows, wrists, metacarpophalangeal joints, proximal interphalangeal joints, and the knees. Moreover, the erythrocyte sedimentation rate (ESR) and the patients' general health (GH) or global disease activity measured on a visual analogue scale (VAS) were considered in the DAS28 calculation formula [17].

(1) $$\begin{array}{c} \text{DAS }28=0.53938\times \text{sqrt}\left(\text{RAI}\right)+0.06465\times \left(44\text{SJC}\right)+0.330\times \ln\left(\text{ESR}\right)+0.00722\times \text{GH}. \end{array}$$

The nutritionist called at the end of each week to assess the patient's compliance to the prescribed diets. Patients with high compliance (>80%) were considered in the final analysis.

### 2.4. Statistical Section

According to the previous study which compared MD and regular diet in DAS 28 [16], the sample size was calculated using the following formula. Fifty-seven patients per group were determined considering 20% dropout.

(2) $$\begin{array}{c} n=\frac{2{\left(Z1-\left(\alpha /2\right)+Z1-\beta \right)}^{2}\;{\left(S2-S1\right)}^{2}}{{\left(\mu 2-\mu 1\right)}^{2}}. \end{array}$$

Statistical analyses were performed using IBM SPSS Statistics, version 25 (IBM Corp, Chicago IL, USA). Qualitative variables were analyzed by the chi-square test. The univariate analysis of variance (ANOVA) test was used to adjust the significant baseline variable's effect on the outcomes. *P* < 0.05 in all comparisons was considered significant.

## 3. Results

**Figure 1:**
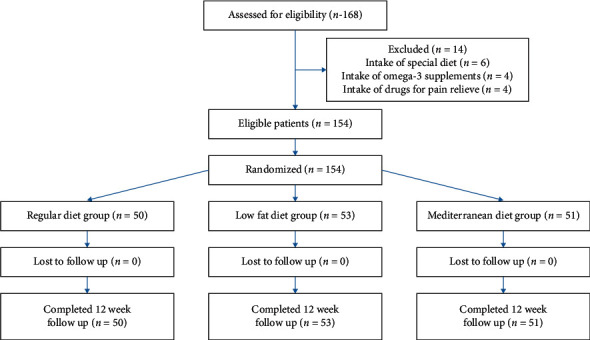
CONSORT flow diagram of the study from baseline up to the end.

The flowchart of the study is shown in Figure 1.

Patient's characteristics before the study enrollment are given in Table 1. There was a significant difference in weight, serum ESR, swollen joints, and VAS among the groups. Patient's weight was significantly higher in the LF-HC and MD groups compared to the controls (*p*=0.02 and *p* < 0.001, respectively). However, weight of participants had no significant difference between the LF-HC and MD groups, significantly. The number of swollen joints was significantly higher in MD compared to the LF-HC and control groups (*p*=0.01 and *p*=0.04, respectively). The number of joints with tenderness had no significant difference among the groups. The serum ESR level was significantly higher in the controls than in the MD group (*p*=0.04). However, no significant difference was seen between the LF-HC and the MD groups. The total score of DAS28 had no significant difference among the groups. Patient's calorie and macronutrient intake including carbohydrate, protein, and fat had no significant difference among the groups (*p* > 0.05).

After 12 weeks of intervention, weight of participants significantly decreased in the LF-HC and MD groups, but no change was seen in the controls. Weight loss was significantly higher in the MD and LF-HC dietary groups than the controls (*p* < 0.001 and *p* < 0.001, respectively). However, the weight changes had no significant difference between the MD and LF-HC dietary groups. Serum ESR level was significantly decreased in MD compared to the LF-HC and controls, at the end of the study (*p*=0.02 and *p* < 0.001, respectively). Moreover, serum ESR was significantly decreased in the LF-HC group compared to the controls (*p*=0.02). Serum ESR changes were significantly higher in MD than in the LF-HC dietary group (*p*=0.007) and the controls (*p* < 0.001). Moreover, serum ESR was significantly decreased in the LF-HC dietary group compared to the controls (*p*=0.007). DAS 28 was significantly decreased at the end of the study. DAS 28 score changes were significantly higher in the MD group than the LF-HC (*p*=0.02) and controls (*p*=0.001). However, there was no significant difference between the LF-HC and the controls in DAS 28 changes at the end (*p* > 0.05). VAS was significantly decreased after 12 weeks of intervention in the LF-HC and MD groups compared to the controls (*p*=0.001, and *p*=0.05). However, the VAS changes had no significant difference between the MD and LF-HC dietary groups (*p* > 0.05) (Table 2).

Adjusting for the baseline variables, the type of diet and serum ESR level showed significant effects on DAS 28. Dietary intervention decreased DAS 28 by 76% (95% CI = −0.45, −0.2; *p*=0.03) after 12 weeks. Serum ESR level at the baseline showed 99.8% effect on DAS 28 at the end (95% CI = 0.014, 0.035; *p* < 0.001). The number of swollen joints at the baseline increased DAS 28 by 78% (95% CI = 0.06, 0.18; *p* < 0.001). Other variables had no significant effect on total DAS 28.

## 4. Discussion

In the present feeding trial on RA patients, the weight loss had no significant difference between the MD and LF-HC dietary groups. Serum ESR was significantly decreased in MD compared to LF-HC and controls after 12 weeks of intervention. Serum ESR changes were significantly higher in the MD group than the LF-HC dietary group and the controls. Moreover, serum ESR was significantly decreased in the LF-HC dietary group compared to the controls. DAS 28 was significantly lower in the MD group than in LF-HC and controls. However, there was no significant difference between LF-HC and controls in DAS 28 changes at the end of the study. VAS was significantly decreased after 12 weeks of intervention in the LF-HC and MD groups compared to the controls. However, the VAS changes had no significant difference between the MD and the LF-HC dietary groups. Because of variations in the RA incidence around the world, recently, risk factors other than the genetics and races have been considered [17, 18]. It is hypothesized that other effective factors such as diet and lifestyle are the determinants of RA occurrence and severity [19]. One of the proposed dietary patterns for RA patients is MD. This dietary pattern combines a set of skills, knowledge, practices, and traditions ranging from the panorama to the practice. This dietary pattern involves crops, harvesting, fishing, conservation, processing, preparation, and consumption of food [20]. Daily consumption of olive oil, unrefined cereals, and fresh or dried fruit and vegetables are the main characteristics of MD. Fish, dairy, and meat are recommended in moderate amounts. Condiments and spices, wine, or its infusions are consumed, frequently [20]. Therefore, MD is rich in nutrients and bioactives with anti-inflammatory effects [21]. Because of variations in food industries, religious, and attitudes in different countries, there is a need to restudy this hypothesis in different countries. On the other hand, obesity is a risk factor for inflammation in the body. There is no study to assess whether weight loss has an important role in pain relief or other factors and bioactives in MD lead to pain reduction. In the intervention studies, there is mixed results for pain. The longer duration studies (≥12 weeks) reported the beneficial effects of MD on pain relief in RA patients. However, inconclusive results were reported on DAS 28 [22, 23]. Because of the progressive and degenerative nature of RA, pain scores would increase with time. The present study was limited in the method of dietary adherence which was assessed based on self-declaration of patients. The DAS 28 questionnaire was filled by patients, that is, dependent to their opinion. According to the literature review, no study compared the effects of the MD pattern with the low-fat diet in a weight loss program up to now. Herein, we concluded that MD has a beneficial effect on DAS 28 compared to the low-fat diet in patients with RA, regardless of weight loss.

## 5. Conclusions

The Mediterranean style decreased DAS 28 compared to the low-fat diet in patients with RA, regardless of weight loss. Therefore, this pattern is advisable to decrease disease severity in patients with RA.

## Figures and Tables

**Table 1:** Patient's characteristics before enrollment to the study.

Groups variables	Controls (*N* = 50)	LF-HC (*N* = 53)	MD (*N* = 51)	*P* value
Age, years	59.2 ± 11.6	58.4 ± 10.9	57.7 ± 12.6	0.09
Sex				
Male	33 (66.7)	32 (61)	31 (60)	0.2
Female	17 (33.3)	21 (39)	20 (40)	
Weight, kg	64.9 ± 10.8	70.9 ± 11.1	74.1 ± 12.3	<0.001
BMI, kg/m^2^	26.6 ± 4.4	27.9 ± 4.5	28.5 ± 4.9	0.005
Energy intake, kcal/d	2762.5 ± 197.3	2845.6 ± 200.5	2872.5 ± 205.4	0.8
Protein, g/d	63.5 ± 5.6	67.2 ± 4.8	65.01 ± 6.7	0.7
Carbohydrate, g/d	320.5 ± 11.9	318.6 ± 15.4	345.6 ± 9.8	0.09
Fat, g/d	110 ± 5.7	122.4 ± 6.5	118.9 ± 5.6	0.2
MUFAs, g/d	34.6 ± 5.4	43.5 ± 9.7	40.9 ± 7.6	0.08
PUFAs, g/d	44.9 ± 9.5	46.2 ± 8.7	46.6 ± 9.1	0.9
SFAs, g/d	30.5 ± 6.8	32.7 ± 7.5	31.4 ± 6.9	0.3
Swollen joints	1.1 ± 2.6	0.95 ± 1.9	2.4 ± 3.5	0.01
Tenderness joints	7.4 ± 4.2	7.35 ± 4.2	6.2 ± 4.4	0.24
ESR, mm/h	25.3 ± 16.9	21.3 ± 18.03	19.7 ± 11.6	0.08
DAS 28	3.8 ± 0.91	3.5 ± 0.88	3.6 ± 0.92	0.2
VAS	5.6 ± 1.4	4.7 ± 1.5	5.1 ± 1.6	0.006

LF-HC, low-fat high-carbohydrate diet; MD, Mediterranean diet; BMI, body mass index; MUFA, mono unsaturated fatty acids, PUFAs, poly unsaturated fatty acids; SFAs, saturated fatty acids; ESR, erythrocyte sedimentation rate; VAS, visual analogue scale; DAS 28, disease activity score 28.

**Table 2:** Mean difference and patient's characteristics after 12 weeks dietary intervention.

Groups variables	Controls (*N* = 50)	LF-HC (*N* = 53)	MD (*N* = 51)	*P* value
Weight, kg				
12 weeks	65 ± 10.5	68.5 ± 10.1	71.2 ± 10.3	0.01
Mean difference	0.32 ± 1.1	−2.37 ± 1.8^a^	−2.9 ± 3.01^a^	<0.001
Swollen joints				
12 weeks	0.38 ± 1.7	0.73 ± 1.8	1.75 ± 3.1	0.008
Mean difference	−0.73 ± 2.03	−0.21 ± 1.4	−0.73 ± 2.2^b^	0.26
Tenderness joints				
12 weeks	2.07 ± 4.04	3.5 ± 4.67	2.77 ± 4.06	0.2
Mean difference	−5.3 ± 4.8	−3.8 ± 4.7	−3.4 ± 4.7	0.1
ESR, mm/h				
12 weeks	24.66 ± 16.4	16.87 ± 13.7	9.23 ± 10.3	<0.001
Mean difference	−0.65 ± 2.4	−4.4 ± 7.9^c^	−8.5 ± 5.6^b^	<0.001
DAS 28				
12 weeks	2.9 ± 1.05	2.67 ± 1.05	2 ± 1.1	<0.001
Mean difference	−0.88 ± 0.86	−0.84 ± 0.98	−1.5 ± 3.01	<0.001
VAS				
12 weeks	5.15 ± 1.6	3.96 ± 1.4	4.4 ± 1.7	0.001
Mean difference	−0.51 ± 0.65	−0.74 ± 0.7	−0.81 ± 0.8	0.1

BMI, body mass index; MUFA, mono unsaturated fatty acids, PUFAs, poly unsaturated fatty acids; SFAs, saturated fatty acids; ESR, erythrocyte sedimentation rate; VAS, visual analogue scale; DAS 28, disease activity score 28. ^a^Mean change was significantly higher in MD and LF-HC than controls. ^b^Mean change of serum ESR was significantly decreased in MD than LF-HC and controls. ^c^Mean change of serum ESR was significantly lower in the LF-HC group than controls.

## Data Availability

The data used to support this study are available from the corresponding author upon request.

## Additional Points

Clinical trial registration: the trial is registered on Iranian clinical trials website under code number: IRCT20200929048876N2. What is known? Weight loss decreases the inflammation in the body. Inflammation is a trigger factor for rheumatoid arthritis activity. Mediterranean diet showed inconclusive results on disease activity. Moreover, there is no study to compare the effect of Mediterranean with low-fat diet on disease activity score in patients with rheumatoid arthritis. What is new? Type of dietary pattern is more important than weight loss. Disease activity decreased in patients received Mediterranean diet, regardless of weight loss. Higher fruits, vegetables, low-fat dairies, vegetable oils, and lower red meat, sweets, and saturated fatty acids are advisable to patients with rheumatoid arthritis.
